# *In-situ* tensile testing of ZrCu-based metallic glass composites

**DOI:** 10.1038/s41598-018-22925-2

**Published:** 2018-03-15

**Authors:** H. C. Sun, Z. L. Ning, G. Wang, W. Z. Liang, S. Pauly, Y. J. Huang, S. Guo, X. Xue, J. F. Sun

**Affiliations:** 1 School of Materials Science and Engineering, Harbin Institute of Technology, Harbin, 15001 China; 2 National Key laboratory for Precision Hot processing of Metals, Harbin Institute of Technology, Harbin, 150001 China; 3 Laboratory for Microstructures, Institute of Materials, Shanghai University, Shanghai, 200444 China; 4 School of Materials Science and Engineering, Heilongjiang University of Science and Technology, Harbin, 150022 China; 5 IFW Dresden, Institut für Komplexe Materialien, Helmholtzstraße 20, D-01069 Dresden, Germany

## Abstract

ZrCu-based bulk metallic glass composites (BMGCs) are well known for their plastic deformability, superior to traditional metallic glasses (MGs), which is attributed to a unique dual-phases structure, namely, the glassy matrix and unstable B2 phase. In the present study, *in-situ* tensile testing is used to trace the deformation process of a ZrCu-based BMGC. Three deformation stages of the BMGC, i.e., the elastic-elastic stage, the elastic-plastic stage, and the plastic-plastic stage are identified. In the elastic-elastic and elastic-plastic stages, the yield strength and elastic limit are major influenced by the volume fraction of the B2 crystals. In the plastic-plastic stage, the B2 phase stimulates the formation of multiple shear bands and deflects the direction of shear bands by disturbing the stress field in front of the crack tip. The deformation-induced martensitic transformation of the metastable B2 phase contributes to the plasticity and work hardening of the composite. This study highlights the formation and propagation of multiple shear bands and reveals the interactions of shear bands with structural heterogeneities *in situ*. Especially, the blocking of shear bands by crystals and the martensitic transformation of the B2 phase are critical for the mechanistic deformation process and illustrate the function of the B2 phase in the present BMGCs.

## Introduction

Bulk metallic glasses (BMGs) have attracted widespread attention owing to their unique mechanical, chemical and physical properties, which makes them one of the most promising structural materials^1–5^. However, strain softening and room-temperature brittleness severely restrict their engineering applications^4^, which is a result of the fast propagation of highly localized shear bands, and leaves no plastic strain deformation in the macro-scale under tension^6^. Therefore, *in-situ* or *ex-situ* formed reinforcement phases were introduced to fabricate composite microstructures, which combine high strength and ductility. Such BMG composites enhance the limited plasticity of BMGs, especially the tensile plasticity^5^. These composite structures included dendrite reinforced Ti-based^7–13^, and globular crystal reinforced ZrCu-based bulk metallic glassy composites (BMGCs)^6,14–19^. However, the mechanical behavior and the deformation mechanisms of BMGCs, such as the BMGCs containing the B2 phase that show obvious work-hardening behavior under tension, have not been well elucidated.

A series of phenomenological theories and concepts, such as free volume^20^, shear transformation zone^21^, and liquid-like zone^22^, as well as flow units^23^ have been proposed to address the plastic mechanism of monolithic BMGs. However, these theories or models hardly describe the deformation mechanisms active in B2 phases reinforced BMGCs on the micrometer scale owing to the applicative length scales.

In the past decades, much attention has been paid on the combination of high strength and excellent ductility for these B2 phase reinforced BMGCs, which has been achieved by means of plastic pre-deformation^17^ and proper alloying additional element^6,24–27^. The precipitation of the B2 phase can influence the stress field that improves the density of shear bands, and optimizes the chemical composition to achieve more stabilized the phase formation of B2^28^. The metastable B2 phase can effectively promote the formation of multiple shear bands, and improve the plastic deformation capability of BMGCs, which is attributed to the complicated stress states of glassy matrix and the secondary phase^17,25,29,30^. In addition, various microstructural factors, such as volume fraction, length scale, and yield strength of the secondary phases, have a great influence on the strain delocalization^31^. What is more, the change in the cooling rate can modify the microstructures of the ZrCu-based BMGCs in terms of crystal sizes and volume faction, which seriously influences the deformation and fracture behavior^32,33^. However, so far the deformation process of B2 phase enhanced BMGCs has not been paid much attention. The exact interaction between the glassy matrix and the crystalline phase is still unclear. The strain controlled martensitic transformation^19^, transformation-induced plasticity (TRIP)^28^ and work-hardening behavior of B2 phase reinforced BMGCs have not been well documented. Motivated by this deficiency, we investigate the deformation process of an B2 phase reinforced BMGCs *in situ*.

In this paper, the microstructural characterization and tensile tests in a Zr-Cu-Al ternary component system, namely Zr_49_Cu_45_Al_6_ (at.%), are conducted to understand the correlation between the microstructure and the tensile properties of BMGCs reinforced with B2 crystals. An *in-situ* observation of the tensile deformation process in SEM is performed to characterize the microstructure evolution with strain. The deformation mechanisms in the glassy matrix and in the crystalline phase during the elastic and the plastic stages are characterized. Finally, also the failure mechanisms of crystalline phases and glassy matrix are discussed.

## Results

### Microstructure of Zr_49_Cu_45_Al_6_ BMGCs

**Figure 1:**
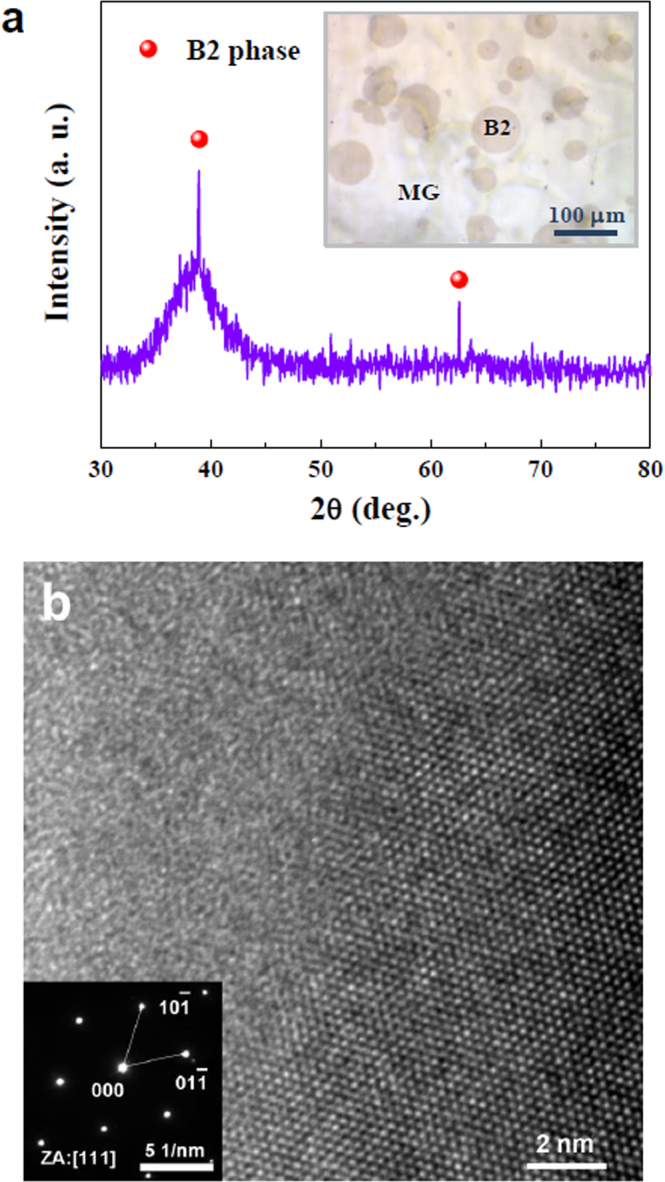
Microstructure of Zr_49_Cu_45_Al_6_ BMGCs. (**a**) XRD pattern of an as-cast Zr_49_Cu_45_Al_6_ rod. The inset shows the corresponding optical micrograph. (**b**) High-resolution bright-field TEM image. Inset shows the corresponding selected area electron diffraction spot of an ordered region which indicates a simple cubic lattice of the crystalline phase.

Figure 1(a) shows a typical XRD pattern of the as-cast Zr_49_Cu_45_Al_6_ alloy, which shows a broad diffuse peak at a diffraction angle of 40°, and two crystalline peaks at 39° and 63°, respectively. The diffuse peak corresponds to the glassy phase. The two crystalline peaks correspond to the lattice planes of B2 the phase in the (110) and (210) directions, respectively. This diffraction pattern clearly suggests a dual-phase microstructure of the Zr_49_Cu_45_Al_6_ BMGC, consisting of the glassy matrix and the B2 phases. The inset of Fig. 1(a) shows an optical micrograph of the BMGC. Circular B2 phases with various diameters between 10 and 100 μm are distributed in the glassy matrix, which is similar to the microstructure of other CuZr-based BMGCs^15,16,25,26^. Figure 1(b) presents a high-resolution TEM image of the BMGC, showing two different phases with a distinct interface. In the left of Fig. 1(b), the microstructure is long-range disordered, which implies the glassy structure of the matrix. On the contrary, the lattice fringes in the right part suggest the presence of a crystal. The inset of Fig. 1(b) further proves that the crystalline phase is the CuZr B2 phase with a simple cubic (Pm-3m) structure.

### Tensile mechanical properties and fracture feature of Zr_49_Cu_45_Al_6_

**Figure 2:**
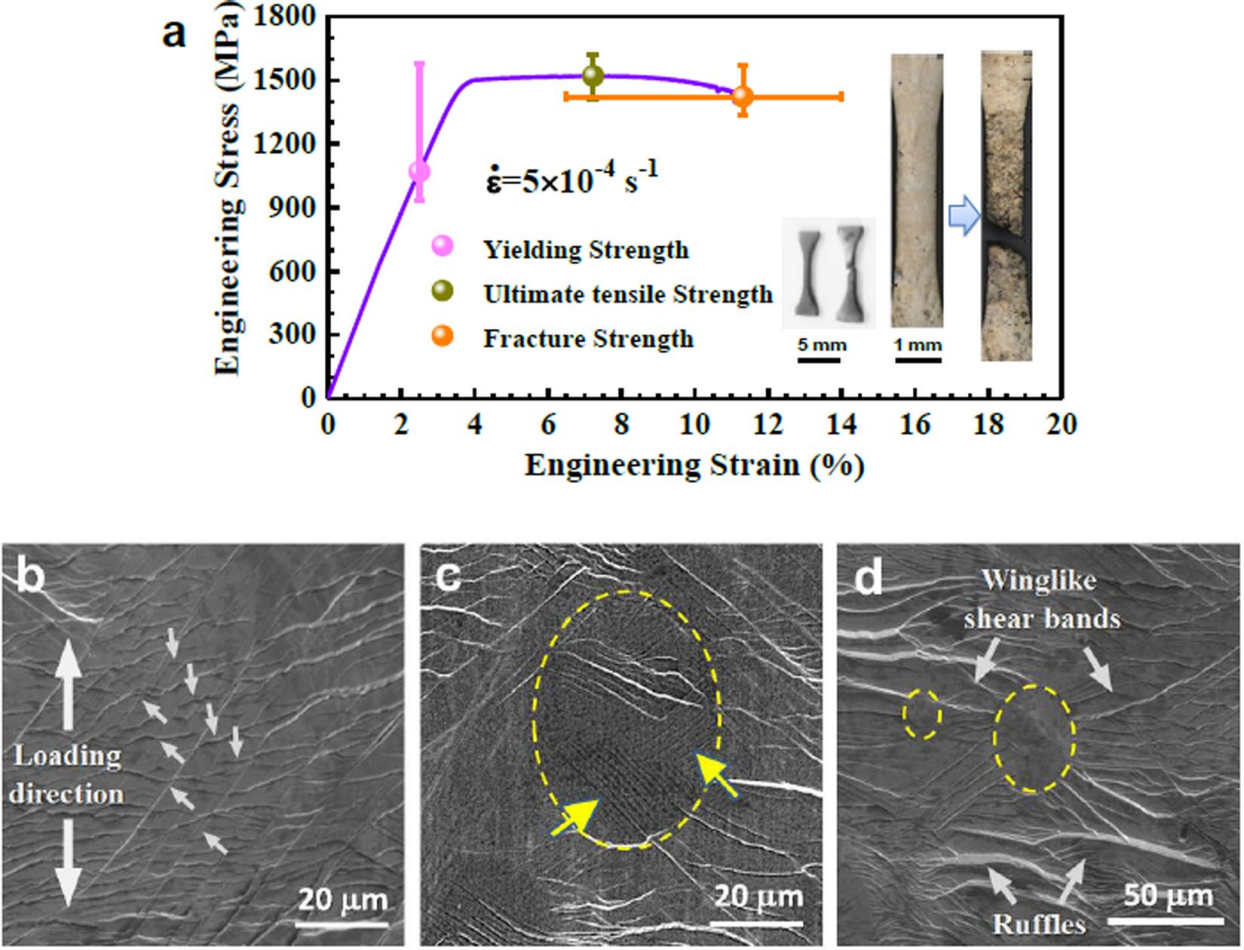
Representative engineering stress-strain curve of Zr_49_Cu_45_Al_6_ BMGCs in tension and the lateral surface morphology of a sample before and after fracture. (**a**) Engineering stress-strain curve. The insets show the micrographs of a specimen before and after tensile test. (**b**) and (**c**) present the lateral surface morphologies of glassy matrix and crystalline phase after tension, respectively. (**d**) presents the cooperative deformation between glassy matrix and crystalline phases.

An engineering tensile stress-strain (σ-ε) curve of the BMGC containing B2 crystals is shown in Fig. 2(a). To ensure reproducibility, the tensile tests were repeated more than five times, which leads to the error bars for the strength values and the strain values indicated in Fig. 2(a). Owing to the influence of the crystalline volume fraction, which ranges from 5% to 25%, the strength values and the strain values fluctuate over a relatively wide range. Therefore, the yield strength and the ultimate tensile strength are 1257 ± 322 MPa and 1516 ± 101 MPa, respectively. The total strain is 10.2 ± 3.7% with a relatively high fracture strength of 1450 ± 119 MPa. It can be seen that the scattering of the ultimate tensile strength and the fracture strength are much smaller than that of the yield strength, and the scattering of the total strain is also very large, which means that the yield strength and the strain of this BMGCs are particularly sensitive to the crystalline volume fraction^32^. The yield strength and fracture strain, as functions of the volume fraction of crystalline phase have been well documented by Pauly *et al*.^19^.

The specimens before and after tensile loading are also shown in the inset of Fig. 2(a). The multiple shear bands distribute over the matrix which implies a non-localized plastic deformation of Zr_49_Cu_45_Al_6_ BMGC. Besides, there are many obvious shear steps in glass and a surface relief in the crystals on the specimen surface after tension, which are shown in Fig. 2(b–d). Figure 2(b) shows the lateral surface of deformed Zr_49_Cu_45_Al_6_ BMGC glassy matrix. Differing from most of BMGs, the BMGC exhibits multiple shear bands indicated by white arrows, and propagating along two main directions, which carry the plastic strain but meanwhile enhance strain softening and promote the instability of glassy matrix^34^. Figure 2(c) shows a deformed B2 precipitate which turns out to be oval and shows some parallel stripes marked with the yellow arrow. It may indicate the occurrence of a martensitic transformation from B2-CuZr to B19’-CuZr during tensile deformation^15,16^. Figure 2(d) shows a local cooperative deformation between the glassy matrix and crystalline phase, where the crystalline phase is surrounded by the glassy matrix with multiple wing-like shear bands aligned perpendicular to the loading direction. In addition, the glassy matrix shows obvious shear steps, which carries the main deformation. Compared to BMGs, the formation and propagation of shear bands seems to be easier for the B2 phase reinforced BMGCs, which can be attributed to the stress concentration fields near the interfaces^35^. The appearance of the multiple shear bands can carry the plastic strain of glassy matrix to avoid catastrophic failure caused by strain localization^18^, while the martensitic transformation of the austenitic B2 phases mediates the plastic instability of glassy matrix and accounts for the hardening of the BMGCs.

### Twinning and martensitic transformation in Zr_49_Cu_45_Al_6_ BMGCs

**Figure 3:**
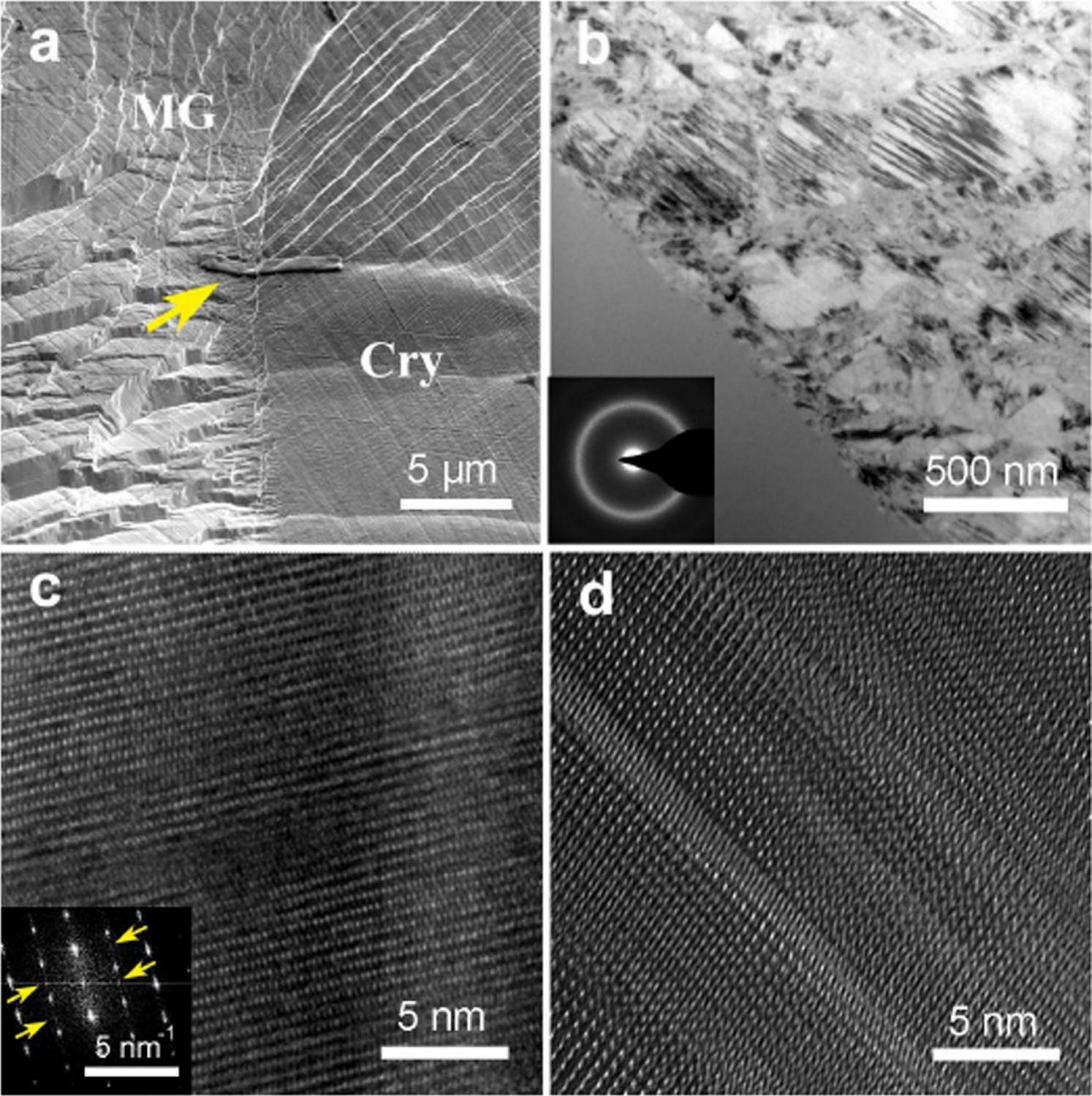
Structural transformation in CuZr-based BMGCs. (**a**) shows the sampling position using FIB. (**b**) TEM micrograph of Zr_49_Cu_45_Al_6_ BMGCs after tension. The inset shows the corresponding electron diffraction pattern of the amorphous part (left below). (**c**) and (**d**) HRTEM micrograph sampling form different parts of the crystal after tension. The inset shows the corresponding electron diffraction pattern transformed by FFT.

To further explore the structural evolution, the microstructures of deformed specimen were observed by TEM. The sampling position, indicated by the arrow (Fig. 3(a)), is the interface between the glassy matrix and the deformed B2 phase just where the edges of the stripes locate, as shown in Fig. 2(c). Figure 3(b) shows the microstructures of both sides of the interface. In the region of the glassy matrix, a homogeneous phase is observed. The selected area electron diffraction (SAED) pattern confirms the glassy nature (the inset of Fig. 3(b)). On the other side of the interface, crystalline phases are observed. The appearance of the interface between the crystals and the glass suggests that the deformation instability preferentially initiates at the unconfined surface, where the shear bands nucleate eventually. The deformed B2 phase shows some obvious parallel stripes on the nanoscale suggesting that localized twinning occurs. Figure 3(c) shows the structure of small strain area where shows little or none stripes in Fig. 3(a). The corresponding diffraction spots of Fig. 3(c) transformed by FFT confirm that twinning occurs in the B2 phase even at small strains. Figure 3(d) shows the structure of large strain area (with stripes) where the martensitic transformation occurs. Comparing with the case subjected to the smaller strain (cf. Fig. 3(c)), the area subjected by the larger strain shows significantly twinning structure, which implies the substructure of martensite is a twin martensite. As the aggravation of strain, twinning of B2 phase is aggravated, which suggests that the twin martensitic transformation is induced by strain. With phase transforming and slipping induced by strain, the B2 phases may eventually form the strips morphology (cf. Fig. 2(c)).

### *In-situ* tensile deformation process

**Figure 4:**
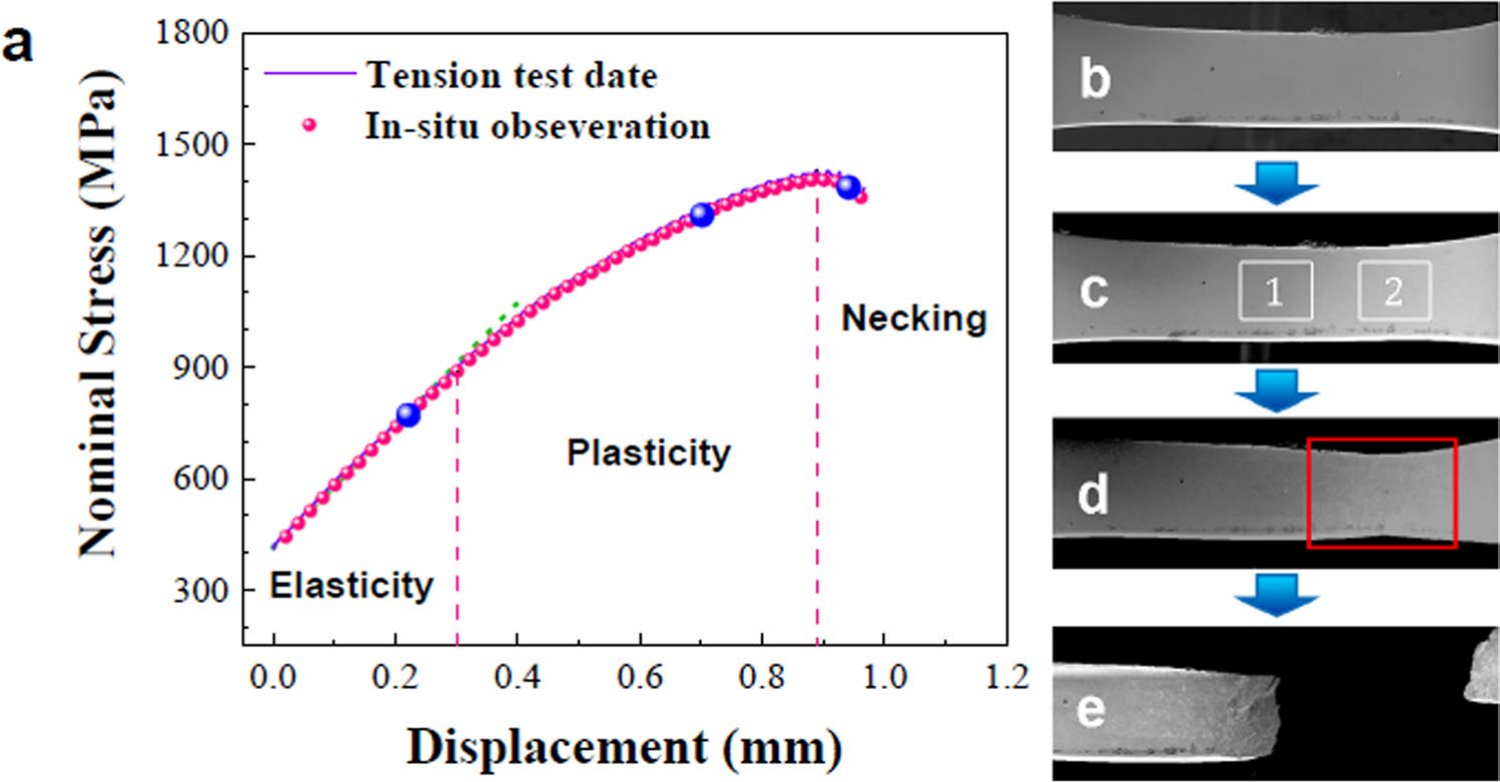
Nominal stress-displacement curve and corresponding stage images of specimen during the *in-situ* tension test.

To better understand the deformation process of the current BMGCs, *in-situ* tensile tests in the SEM were carried out (see Supplementary material). Figure 4(a) shows a nominal stress-displacement curve of an *in-situ* tensile test. Three stages are observed in the stress-displacement curve, i.e., the elastic stage, the plastic stage and the necking stage. The corresponding SEM images are also depicted in the right column of Fig. 4. In the elastic stage (Fig. 4(b)), the BMGC does not show obvious change in the shape. The stress-displacement curve shows linear elastic deformation. Following straining, non-linear deformation occurs, suggesting that irreversible plastic deformation occurs. The corresponding image is shown in Fig. 4(c), in which the initiation and propagation of multiple shear bands are observed. With increasing the density of shear bands, the plastic strain is localized into certain regions and leads to a reduction in the cross-sectional area, meaning the occurrence of an unstable plastic deformation, and that necking becomes more and more pronounced as shown in Fig. 4(d). After necking, the plastic deformation is confined in the necking region, and then causes the final fracture.

**Figure 5:**
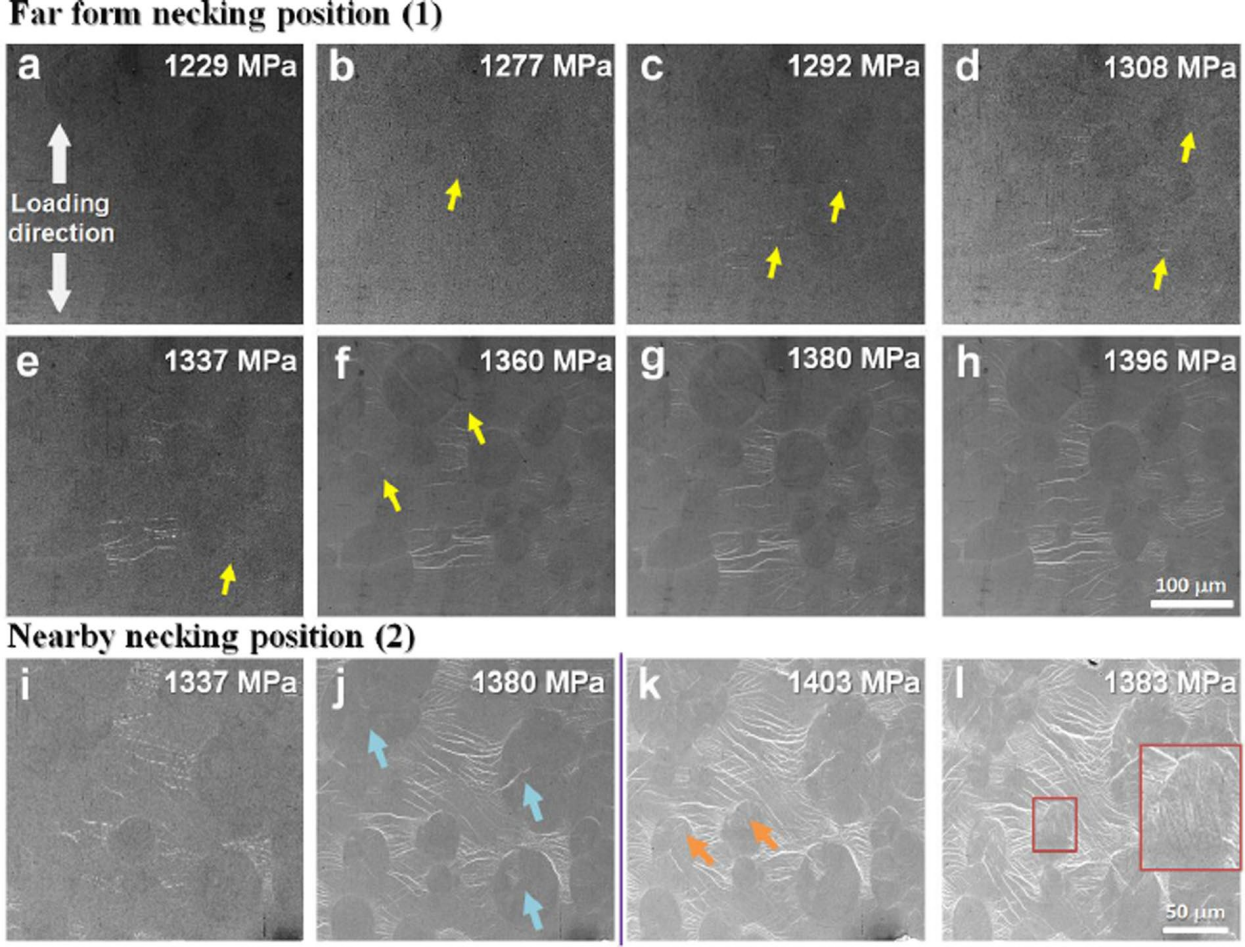
*In-situ* observation of the lateral surface morphology evolution of different regions under tension (see Supplementary material). (**a**–**h**) show the dynamic formation and propagation of multiple shear bands in position (1). (**i**–**l**) shows the dynamic deformation procedure of the crystalline particles before and during necking, respectively.

Based on the *in-situ* observations, position 1 marked in Fig. 4(c) is further enlarged to analyze that the shear bands successively nucleate at the interface of B2 phases and glassy matrix, which is marked by the yellow arrows in Fig. 5(b–f). The shear bands in general form perpendicularly to the loading direction, which is influenced by the stress field around the B2 particles. Similar results have been shown in some BMGs with artificial defects holes that facilitates the nucleation of multiple shear bands^36^. With increasing the plastic strain, more shear bands radially nucleate along the crystal-glass interface. After ongoing straining, multiple shear bands form and propagate towards the neighboring B2 particles as shown in Fig. 5(e and f). Considering the stress field around one inclusion^30^, the distribution of stress field is believed to coincide with the distribution of the multiple shear bands, as shown in Fig. 2(d). This suggests that the propagation of the shear bands is severely influenced by the stress field around the B2 crystals. Since the B2 phase is embedded into the glassy matrix, the continuity of the glassy matrix is broken. The interfaces and structural discontinuities in the glassy matrix may influence the stress transfer, in which the stress concentration is formed and shear bands are initiated. The B2 phase effectively promotes the density of stress concentration sites, and then enhances the formation and the interaction of multiple shear bands, which can significantly stabilize the tensile plastic deformation of BMGCs^29^. On further straining, the shear bands propagate and the stress field in the front of the shear band tip is disturbed by a given B2 particle, which deflects the propagation path of shear bands towards the neighboring B2 precipitate. The elastic energy released due to the shear band propagation can be efficiently absorbed by the plastic strain in the soft B2 phase, leading to the proliferation of shear bands^37^. According to *in-situ* observations, the “blocking effect” of B2 phases can be considered as that the shear bands are trapped by the B2 phases, and the elastic energy for the propagation of shear bands is absorbed by the plastic deformation of the B2 crystals.

Figure 5(i–l) capture the plastic deformation process of position 2 marked in Fig. 4(c), which reflects the necking process. Prior to necking, the crystals do not show any obvious interfacial de-bonding with the glassy matrix. The shape of B2 phase is nearly spherical as shown in Fig. 5(i). With straining, the crystal marked by the arrow in Fig. 5(j) is surrounded by multiple shear bands, and the interface becomes much clearer. Some faint internal strips are formed in the crystalline phase, which implies a martensitic transformation has occurred. In addition, the shape of the crystal becomes elliptical with the long axis being parallel to the loading direction, which suggests a cooperative deformation between the matrix and the crystals. Furthermore, some cracks pointed by the blue arrows also nucleate in the crystalline phase. With increasing strain, the specimen necks and the density of shear bands increases further. The aggravation of crystal deformation causes some new strips to emerge in some precipitates, which mainly appears on the top of ellipses where the largest deformation occurs in the crystals. Thus, the deformation of the crystalline phase is inhomogeneous as shown in inset of Fig. 5(l). The obvious strips always emerge at the large deformation position, implying that the martensitic transformation is the primary mechanism of crystal deformation.

Comparing Fig. 5(e,g) with (i), (j), the number of shear bands at same stress in different position is quite different, which indicates the deformation of the BMGC is locally inhomogeneous. The reason might be the distribution and the size of crystalline phase that influences the local stress field.

### Failure modes

With straining, the specimen necks before fracture. Although the BMGC deforms with multiple shear bands that can effectively prevent the highly localization of shear strain, and then causes the plastic deformation in the glassy matrix, once a shear band propagates without being blocked by crystals, the fracture process of the BMGCs is almost identical to the process in monolithic BMGs. The propagation direction of main shear band is also influenced by the stress field generated by the presence of the secondary phase.

**Figure 6:**
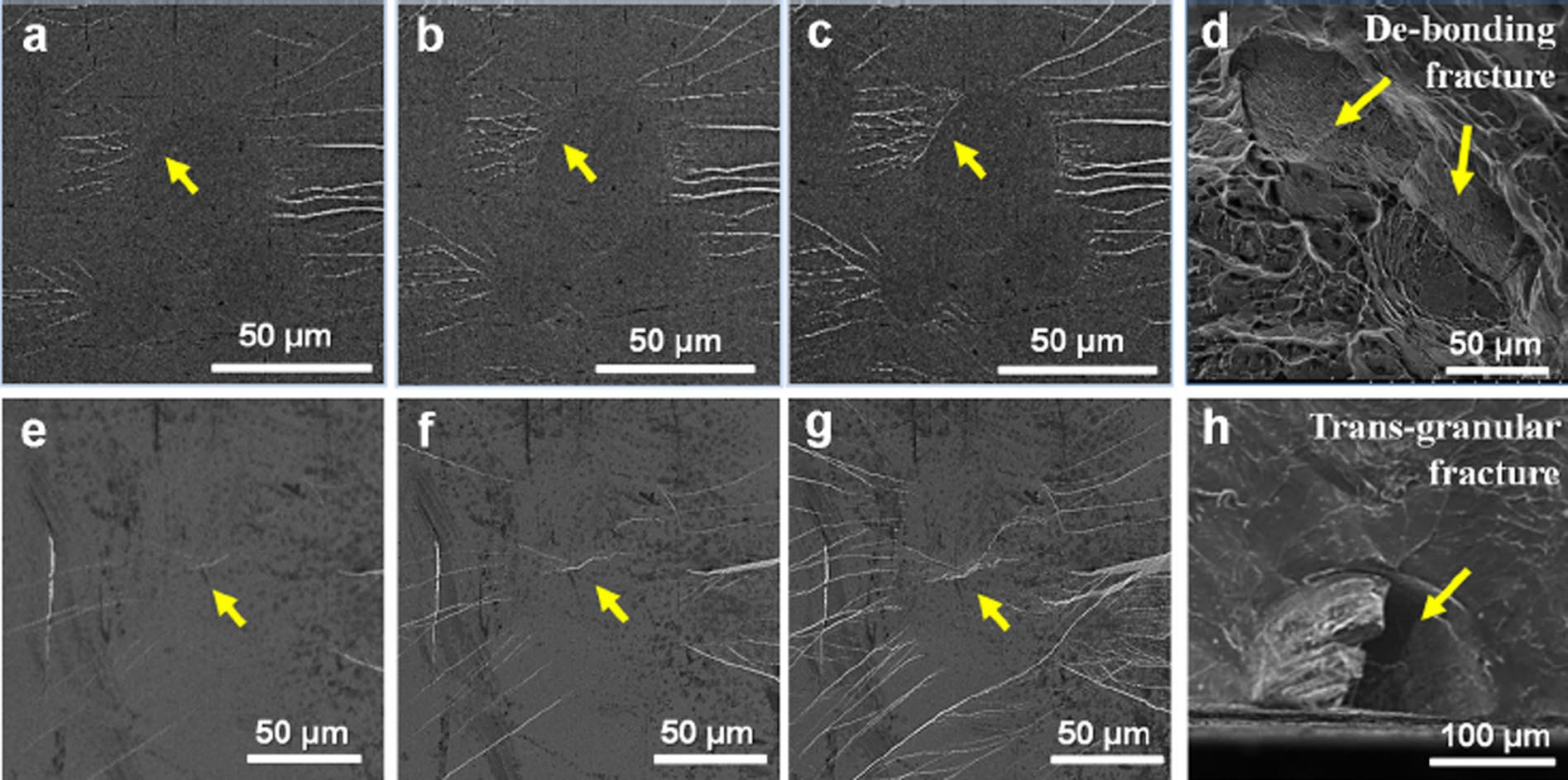
Two fracture paths of BMGCs contenting the B2 phase. (**a**–**d**) present the interfacial de-bonding process and the corresponding morphology. (**e**–**h**) show the transgranular fracture process and its morphology.

Owing to the participation of crystalline phase in fracture process, the CuZr-based BMGCs failure could be classified as two modes based on the morphologies of fracture. Figure 6 shows two possible failure modes and their observations *in situ*. When the deformation of the B2 phase is inhomogeneous in the glassy matrix shown in Fig. 6(a), interfacial de-bonding emerges from the shear bands, in which the stress field is heavily influenced by the interfaces. Then, the deformation favors the propagation of interfacial cracks, which grow and finally coalesce, as shown in Fig. 6(b and c), and finally results in interfacial de-bonding as shown in Fig. 6(d), which is a typical behavior in BMGCs reinforced by B2 phases according to the repeated trials. The slippage and laths structure usually appear in the martensitic transformation of secondary phase, which enables the stress accumulating on the interfaces, and makes it much easier to craze. On the other hand, the crystallographic defects or uncoordinated deformation of the crystals may induce the stress concentration, which then results in cracking. Upon further loading, the crack formation and propagation are shown in Fig. 6(e–g). It can be seen that the inner crack propagates through the interface, and connects with the external multiple shear bands, which finally leads to the trans-granular fracture morphology as shown in Fig. 6(h).

## Discussion

### Tensile deformation process of CuZr-based BMGCs

**Figure 7:**
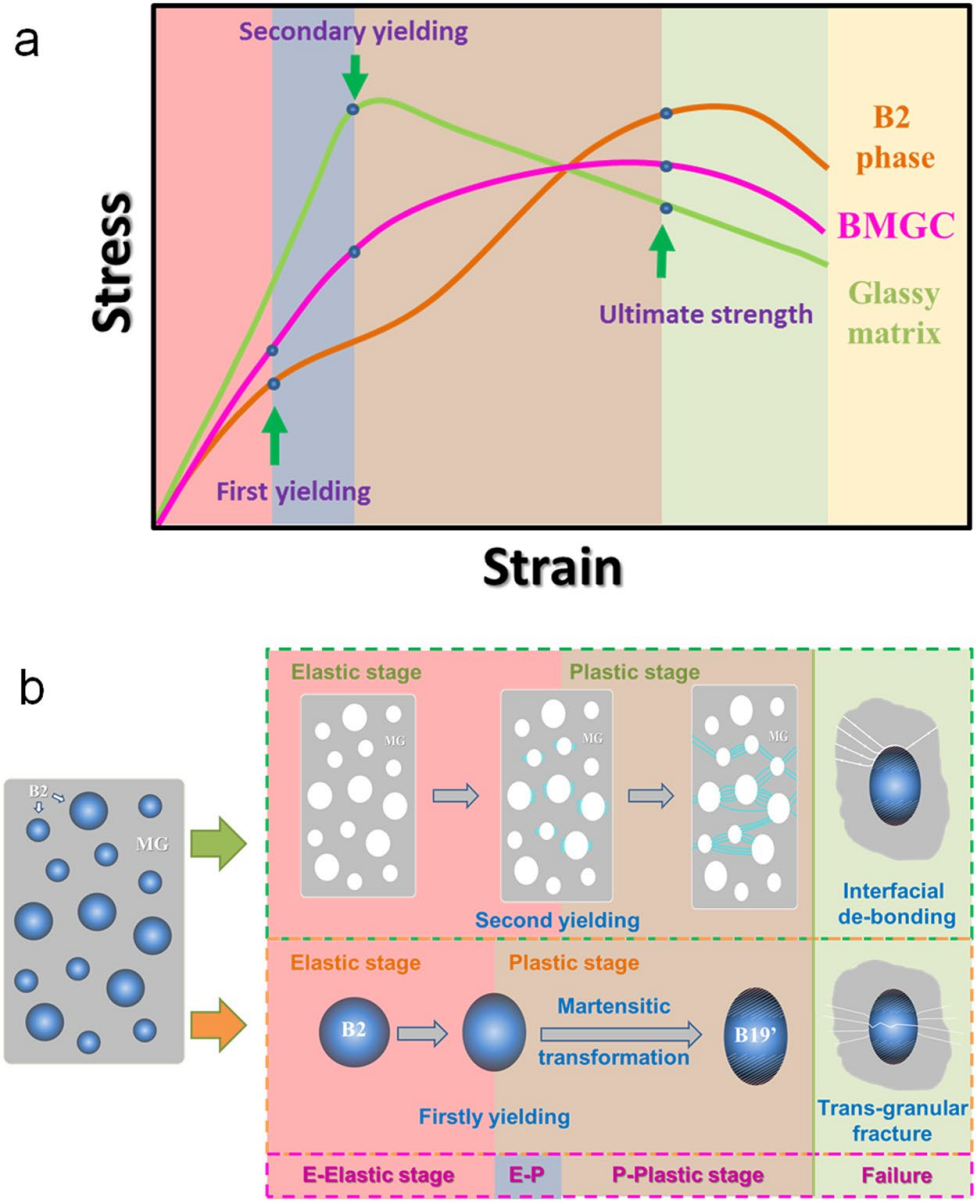
Illustration of deformation processes of CuZr-based BMGCs. (**a**) Schematic of the stress-strain curves of BMGCs and its corresponding B2 phases and glassy matrix. (**b**) deformation processes illustration of B2 phases and glassy matrix in each stage.

Based on the results shown in Fig. 4, and regarding the tensile behavior of two phases, the tensile deformation process of CuZr-based BMGC is sketched in Fig. 7(a). The deformation process is divided into four stages as shown in Fig. 7(b). The first stage is the elastic-elastic stage, in which both phases deform elastically. Further increasing the strain, the B2 phase is plastically strained, and the glassy matrix remains in the elastic range, which is an elastic-plastic stage. In these two stages, no obvious changes in terms of the shape of the specimen (Fig. 4) occur, and the glassy matrix deforms elastically without the formation of shear bands. Upon further straining, both phases deform plastically and the shear bands nucleate and propagate in the glassy matrix. Simultaneously, the martensitic transformation in the B2 phase sets in. In this stage, plastic deformation is stable, corresponding to the plastic region, in which the work-hardening behavior results from the interaction between the glassy matrix softening and secondary-phase hardening. Finally, when the work-hardening effect resulting from B2 phase is reduced, plastic deformation is instable, leading to localized straining in the composite, which usually causes necking or fracture^38^.

### Elastic-elastic deformation of CuZr-based BMGC

In the elastic-elastic stage, coordinated deformation must be activated between the glassy matrix and the B2 phase. The stress-strain relations of the glassy matrix and B2 phases are expressed as^9,11,39^,

1 $$\{\begin{array}{c}{\sigma }_{MG}={E}_{MG}{\varepsilon }_{MG},\,{\sigma }_{MG}\le {\sigma }_{yMG}\\ {\sigma }_{B2}={E}_{B2}{\varepsilon }_{B2},\,{\sigma }_{B2}\le {\sigma }_{yB2}\end{array},$$

where *σ* is the tensile stress, *E* is Young’s modulus, *ε* is the elastic strain, and *σ*_*y*_ is the tensile yield stresses. The subscripts of MG and B2 represent the metallic glassy matrix and the B2 phase, respectively.

The Poisson’s ratios of the glassy matrix and the B2 phases are 0.373 and 0.385, respectively^14^, which are very similar. The effective Young’s modulus, *E*_*BMGC*_, of the dual-phase composite can be estimated as^40^:

2 $${E}_{BMGC}={E}_{MG}[1+\frac{{f}_{v}({E}_{B2}-{E}_{MG})}{(1-{f}_{v})\beta ({E}_{B2}-{E}_{MG})+{E}_{MG}}],$$

where *f*_*v*_ is the volume fraction of the B2 phases, *β* is the material constant of Eshlby’s S-tensor for spherical inclusions^39,41^, which is calculated by $\beta =\frac{2}{15}\cdot \frac{4-5{\nu }_{MG}}{1-{\nu }_{MG}}$ (where *ν*_*MG*_ is the Poisson ratio of the glassy matrix.).

To determine the phase that will yield first with increasing the load, the average stress concentration factors of the glassy matrix, *c*_*MG*_ and B2 phase, *c*_*B2*_ are calculated to be^42^:

3 $${c}_{MG}=\frac{\beta ({E}_{B2}-{E}_{MG})+{E}_{MG}}{[{f}_{v}+(1-{f}_{v})\beta ]({E}_{B2}-{E}_{MG})+{E}_{MG}},$$

4 $${c}_{B2}=\frac{{E}_{B2}}{[{f}_{v}+(1-{f}_{v})\beta ]({E}_{B2}-{E}_{MG})+{E}_{MG}}.$$

According to the previous results^14,15^, *β* is 0.454 and *c*_*MG*_ is almost same to *c*_*B2*_ because of the similar compositions in the two phases. The yield strength of the B2 phase is much lower than that of the glassy matrix. Therefore, ${\sigma }_{yMG}/{c}_{MG} > {\sigma }_{yB2}/{c}_{B2}$. Hence, we can infer that the ductile B2 phase yields first during the tensile test^39,42^, and the effective Young’s modulus of the B2 phase in the elastic-elastic deformation stage is slightly lower than that of the glassy matrix, as sketched in Fig. 7.

At the early stage of elastic deformation, the elastic strain of the composite, *ε*_*BMGC*_, is equivalent to the elastic strains in the constituting phases, i.e., *ε*_*BMGC*_ = *ε*_*MG*_ = *ε*_*B*2_. Using the first-order approximation, the axial stress of the dual-phase composite can be evaluated, which is highly dependent on the stresses of glassy matrix and B2 phases:

5 $${\sigma }_{BMGC}={f}_{v}{\sigma }_{B2}+(1-{f}_{v}){\sigma }_{MG},$$

where *σ*_*BMGC*_ is the stress of the composite. The boundary conditions are not considered in present study. Thus, the simple approximation is adopted.

With further straining, the soft B2 phase reaches the elastic limit that can be calculated from the Young’s modulus and the yield strengths of glassy matrix and B2 phases^14,15^, namely ${\varepsilon }_{e}={\varepsilon }_{B2}=\frac{{\sigma }_{yB2}}{{E}_{B2}}\approx 0.6 \%$. At the elastic stain limit of the B2 phase, ε_B2_ = ε_MG_ = ε_BMGC_. Based on Eqs (1) and (5), the constitutive relation of first yielding strength of composites and volume fraction of secondary phases can roughly be expressed as:

6 $${\sigma }_{e}=-({E}_{MG}-{E}_{B2}){\varepsilon }_{e}{f}_{v}\,+{E}_{MG}{\varepsilon }_{e},$$

where *σ*_*e*_ is the first yielding strength of the composite. (*E*_*MG*_ − *E*_*B*2_)*ε*_*e*_ and *E*_*MG*_*ε*_*e*_ are constants, which imply that *σ*_*e*_ is linearly depended on the volume fraction of the B2 phase in a small range.

When the B2 phase yields, the nonlinear stress-strain relation of the B2 phase causes the deformation of the entire composite to deviate from the linear behavior, in which the volume fraction of the B2 phase is dominant according to Eq. (5). A larger B2 phase volume fraction means that the deformation behavior becomes much closer to the nonlinear relation, which implies that the volume fraction of the B2 phase can significantly influence the second yield of BMGCs. The first yielding actually occurs when the strain is about 0.6% even though the near-linear deformation can hardly be identified when the volume fraction is too small.

### Slip, twinning and martensitic transformation of the B2 phase

**Figure 8:**
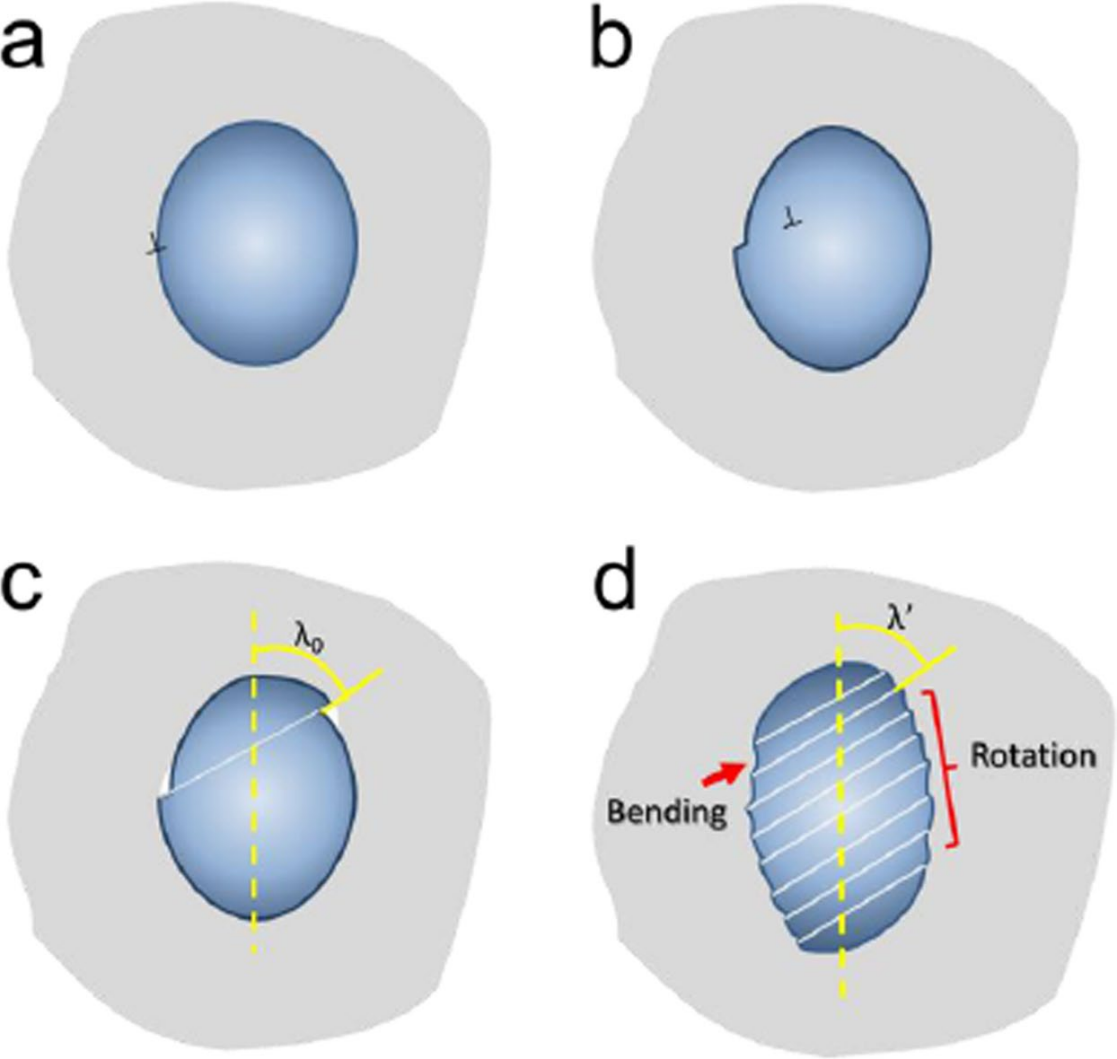
Schematic diagram of deformation mechanism of a single B2 phase. (**a**–**c**) show a whole process of dislocation slip in crystal. (**d**) shows the diagrammatic bending and rotation in crystal.

After the elastic-elastic stage, the B2 phase yields first. Owing to the phase transformation of B2 phase during the tension, the stress-strain schematic of B2 phase behaves “M”-mode process as shown in Fig. 7. The B2 phase can be regarded as a single crystal clamped by the glassy matrix to simplify the analysis (Fig. 8). A single crystal resolved shear stress, *τ*, can be expressed as:

7 $$\tau =\frac{F}{A}\,\cos \,\varphi \,\cos \,\lambda ,$$

where *F* is the tensile force, *A* is the transverse cross-sectional area of the crystal, *ϕ* is the angle between the normal direction of slip plane and the tensile direction, and *λ* is the corresponding angle between the slip direction and the tensile axis. For the B2 phase, the various slip systems are usually oriented differently. One of these slip systems experiences the greatest resolved shear stress, on which plastic deformation mediated by dislocations initiates.

In the elastic-elastic stage, the glassy matrix and the B2 crystals deform under different stress states. At the same strain, the different Poisson’s ratios lead to an increased stress concentration at the interface between the two phases, which initiates the dislocations from the interface of the B2 phase as shown in Fig. 8(a). Then, the dislocation is easy to slip to the other interface in crystal, which makes the crystal deforming plastically (Fig. 8(b and c)). Thus, plastic deformation of the B2 phase is the transmitted bourne by dislocation glide. When one slip system is activated, the work-hardening effect is relatively low. With straining, multiple shear slip and significant dislocation motion commence. The glassy matrix constrains the deformation of the B2 phase during tension, the elongation of the B2 particles in the loading direction and the shrinkage in the transverse direction are impeded. This results in a rotation in the angle between the tensile axis and the normal direction of the slip plane, and, consequently, the slip direction deviates from its original value as shown in Fig. 8(d). Concurrently, other slip systems also begin to rotate to respond to the permanent deformation and the dislocation interaction, which thus enhances the work-hardening effect dramatically. In addition, under the grabbing effect of the interface, bending of the crystal boundary may take place, which can improve the shear-band formation in the glassy matrix. With increasing strain, the work-hardening rate is reduced due to saturation, suggesting that the deformation of the B2 crystals is dominated by a softening effect.

Although dislocation glide is an importance mechanism of plastic deformation in crystals (cf. Fig. 5), twinning is also a way by which permanent shape changes can be realized especially in this simple cubic metal. Comparing Fig. 5(c and d), the density of twins in the obviously deformed part of the specimen is greater than in the other parts. Twinning can reorient the crystals in a way, in which the applied stress mostly results in slip. It can be surmised that the deformation twinning can serve to facilitate slip.

The martensitic transformation is the outcome of cooperative slip and twinning, in which the slip provides the lath structure, and deformation twinning and stacking faults are responsible for the substructure of the martensite.

### Multiple shear bands in BMGCs

In both the elastic-elastic and elastic-plastic stages, no cracks or shear bands are initiated in the glassy matrix, which is consistent with the results from our *in-situ* tensile tests (Fig. 4). Once the glass matrix yields, the plastic-plastic stage commences, in which the plasticity of the composite is composed of two parts, i.e., the plastic deformation of the glassy matrix and of the B2 phase.

In contrast to monolithic BMGs, the internal stress state in the BMGCs is much more complicated during loading because the stress concentration in the secondary phase is influenced by many factors, such as the grain size, the particle size, the distance between neighboring particles and so on^7^. The nucleation and propagation of shear bands in the glassy matrix are an inherently highly transient and inhomogeneous process^36^, which makes it so difficult to observe the origin of shear banding in a sample. In this case, the *in-situ* observations of tensile deformation may provide some important clues to discover the plastic origination.

The dislocation motion occurs in the B2 phase. The dislocation motion is stopped by the interface between the glassy matrix and the B2 phase, on which the pile-up of dislocations will lead to a local stress concentration. This stress concentration at the interface, in turn, will initiate shear banding in the glassy matrix. The precipitation of crystals suspends the continuum of the glassy matrix. This discontinuity can dominate the origin of shear bands in BMGCs^36^. When a propagating shear band enters the stress-affected zone near a B2 precipitate, the stress field around the tip of the shear band is deflected. It then propagates towards the interface of the B2 particle, which releases the elastic energy at the interface and contribute the local stress concentration which activates the slip and twinning behavior in the B2 phase. This is the “blocking effect” that the B2 phase exerts on the shear bands.

### Failure

In the plastic-plastic stage, the work-hardening behavior of the composite governed by the B2 phase is weakened. Once the work-hardenability of the B2 phase becomes weaker than the softening effect from the glassy matrix, the deformation of the composite will be dominated by shear banding, in which the stress within the localized necking part continues to increase. Thus, the resistance to localization will be decreased, and softening in the composites occurs, which corresponds to the necking stage, as shown in Fig. 3, as well as to the softening stage, as shown in Fig. 5. The fracture of the B2 phase in the BMGCs is associated with the angle between the slip planes and the loading axis. For instance, when B2 crystals rotate in such a way that the external stress is applied along the [001] direction, the slip [010] direction on the (100) slip plane cannot be activated, and fracture occurs in the B2 phase, which is shown as Fig. 6(e–h).

## Conclusions

In conclusion, Zr_49_Cu_45_Al_6_ BMGCs with a uniform distribution of B2 precipitates exhibits an excellent combination of tensile strength and plastic strain at room temperature. Both entities are determined by the volume fraction and the length scale of the B2 particles, which might provide a useful measure to tune the mechanical properties, such as the ductility and strength, of BMGCs by controlling the volume fraction and the grain distribution of B2 phases.

The deformation process of the BMGCs includes three stages corresponding to an elastic stage, a hardening stage and a softening stage. In the elastic stage, the elastic limit and yield strength are mainly governed by the volume fraction of the constituent phases. The hardening stage could be divided into an elastic-plastic and a plastic-plastic deformation regime, respectively. In the elastic-plastic regime, elastic deformation of the glassy matrix renders the plastic deformation of the crystalline phases more homogeneous, which simultaneously generates the work-hardening behavior. In the plastic-plastic regime, the coordinated plastic deformation occurs between the glassy matrix and the crystalline phase. The shear bands nucleate and propagate along the interface that is perpendicular to the loading direction, and then branch to form multiple shear bands. Deformation of the B2 phase is confined by the glassy matrix until it induces the martensitic transformation in the B2 crystals, which also causes work hardening. Once, multiple shear bands propagate and de-bonding at the interface between crystal and glass commences, the work-hardening effect of the crystalline phases weakens, and softening of the glassy matrix dominates deformation of the BMGCs. Eventually, this causes softening and necking.

The present study provides a fundamental understanding of the deformation mechanisms on tension acting in BMGCs, which contain B2-CuZr precipitates. Our findings are important for developing and for designing work hardening dual-phase BMGCs with excellent ductility and high strength.

### Experimental methods

Zr_49_Cu_45_Al_6_ ingots were fabricated by arc melting of mixtures of the elements (Zr, Cu and Al of 99.99% in purity) in a Ti-gettered argon atmosphere. To guarantee the chemical homogeneity, each ingot was melted four times with an arc melting current of 250 A. After being melted, the ingot was suction-cast into a copper mold to form a rod with a diameter of 4 mm and a length of 45 mm.

The phase formation was characterized by X-ray diffraction (XRD) with Cu-Kα using an Empyrean diffractometer. The fractured BMGCs, after the tensile tests, were additionally investigated by a micro-region X-ray diffraction using a D/max-2500 V+ diffractometer. The microstructures of the as-cast samples and the samples after the tensile tests were examined by means of a Tecnai F20 field-emission transmission electron microscopy (TEM). The TEM samples were prepared using twin-jet electro polishing or focused ion beam (FIB) in a FEI-600i FIB/SEM dual-beam system. The fracture morphologies were observed in a HITACHI SU-1500 scanning electron microscope (SEM).

The tensile specimens were electric discharge machined from the middle and lower part of the as-cast rods. All tensile specimens were ground and finally electrolytic polished to remove all traces from mechanically grinding. The tensile tests were conducted in an Instron CMT 5205 testing machine at room temperature with an initial strain rate of 5 × 10^−4^ s^−1^. The *in-situ* tension tests were conducted in an Apollo 300 SEM with a micro tensile module at a constant speed of 0.05 mm/min.

## Electronic supplementary material

Overview of the whole process

Formation and propagation of multiple shear bands

Deformation and martensitic transformation of crystals

Deformation and martensitic transformation of crystals
